# Long-term variation of ^90^Sr and ^137^Cs in environmental and food samples around Qinshan nuclear power plant, China

**DOI:** 10.1038/s41598-021-00114-y

**Published:** 2021-10-22

**Authors:** Yiyao Cao, Zhixin Zhao, Peng Wang, Shunfei Yu, Zhongjun Lai, Meibian Zhang, Xiangjing Gao, Yaoxian Zhao, Zhiqiang Xuan, Hong Ren, Dongxia Zhang, Xiaoming Lou

**Affiliations:** 1 Department of Occupational Health and Radiation Protection, Zhejiang Provincial Center for Disease Control and Prevention, Hangzhou, 310051 Zhejiang China; 2 Hangzhou Hospital for the Prevention and Treatment of Occupational Disease, Hangzhou, 310051 Zhejiang China; 3 National Institute of Occupational Health and Poison Control, Chinese Center for Disease Control and Prevention, Beijing, 100050 China

**Keywords:** Environmental chemistry, Environmental monitoring

## Abstract

Environmental radioactivity monitoring in the surroundings of nuclear facilities is important to provide baseline data for effective detection in case of any radioactive release in the region. In this work, we report for the first time the long-term monitoring data of ^137^Cs and ^90^Sr in environmental and food samples around Qinshan nuclear power plant in 2012–2019. The distribution levels, temporal variations and source terms of ^137^Cs and ^90^Sr in the investigated samples were discussed. The annual effective dose (*AED*) for the local population from the ingestion of foods was also evaluated. Peak values of ^90^Sr and ^137^Cs concentrations and ^137^Cs/^90^Sr activity ratio were observed in total atmospheric deposition in 2016 and some water and food samples in the following years. This seems to be associated to an additional radioactive input, mostly likely from the operational release of a local facility. This demonstrates that ^90^Sr and ^137^Cs, especially the ^137^Cs/^90^Sr activity ratio, are sensitive indicators for detecting potential radioactive releases. Nevertheless, overall ^90^Sr and ^137^Cs activity concentrations measured during 2012–2019 in this work were at the background levels with average *AED* far below the internationally permissible limit and recommendation.

## Introduction

Over the past decades, the proportion of energy sourced from nuclear power has increased rapidly worldwide. China has vigorously developed nuclear power in recent years^1^. Systematic monitoring of important anthropogenic radionuclides is crucial for revealing sources and transport pathways in case of sever accidental or deliberate releases of radioactive pollutants. Among the radionuclides released from the operation of nuclear facilities and nuclear accidents, the two high-yield fission products ^90^Sr (t_½_ = 28.79 y) and ^137^Cs (t_½_ = 30.17 y) are recognized as the most important from the radiological perspective^2^. Due to their relatively long half-lives, ^90^Sr and ^137^Cs can be preserved in terrestrial and marine systems for a long time once entering the environment. Under natural environmental conditions, ^137^Cs and ^90^Sr mainly enter the human body through the food chain and respiration. Both ^90^Sr and ^137^Cs have long biological half-lives in the human body^3–8^, therefore it is important to continuously monitor ^90^Sr and ^137^Cs in environmental and food samples, especially those from the surroundings of nuclear facilities, to ensure the radiological safety of individuals and the environment. In contrast to ^137^Cs, long-term monitoring data for ^90^Sr activity in environmental and food samples worldwide are sparse. This fact is mainly attributed to the long and tedious sample preparation and measurement procedures for ^90^Sr.

The Qinshan nuclear power plant (QNPP) is the first nuclear power plant in China and officially commenced commercial operation in December 1991. The QNPP is a multi-unit nuclear power plant, and consists of three phases NPPs operating two heavy water reactors (HWRs) and seven pressurized water reactors (PWRs) with a total installed capacity of ca. 6.5 GW. Artificial radionuclides such as ^3^H and ^14^C in the vicinity of QNPP have been investigated to a very limited extend during the past decades^9,10^. Studies on primary fission products in environmental and foods samples, especially long-term systematic studies have not been reported so far. In this work, we report for the first time a long-term observation of ^137^Cs and ^90^Sr in environmental and food samples collected around QNPP during 2012–2019. The distribution levels, temporal variations and source terms of ^137^Cs and ^90^Sr in the study region were investigated. The annual effective dose was estimated for the local public based on our measurement data.

## Materials and methods

### The study region and sample collection

**Figure 1:**
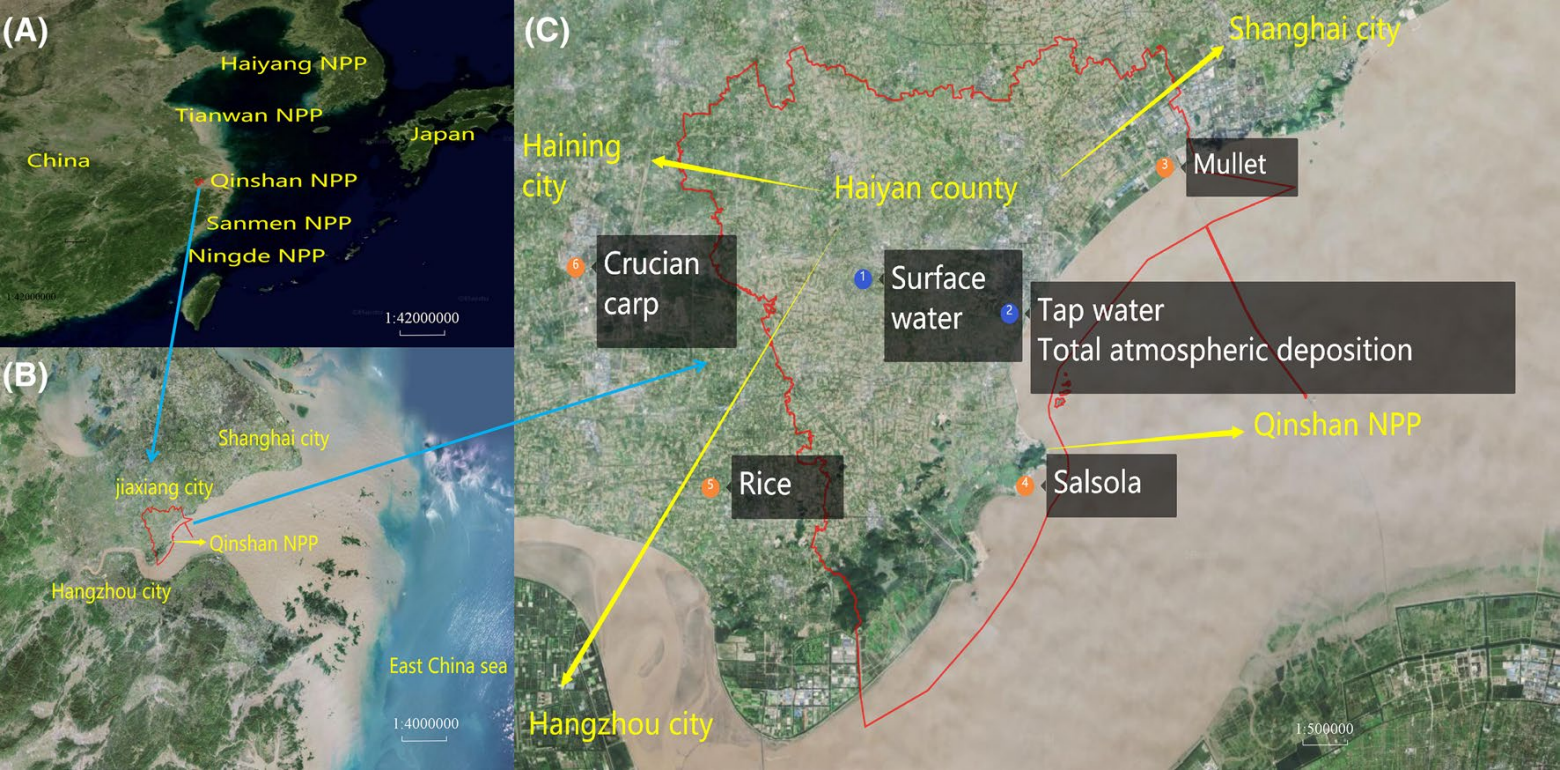
Map showing the geographical location of QNPP (**A**, scale: 1:42,000,000) and (**B**, scale: 1:4,000,000), and sampling sites in the surroundings of QNPP marked with filled circles and numbers (**C**, scale: 1:500,000). The map was produced using software MapInfo Professional using data from https://bajiu.cn/ditu/.

The QNPP is located in Haiyan County, Jiaxing City, Zhejiang Province in China, close to Hangzhou Bay in the East China Sea, 130 km away from Shanghai, and 93 km away from Hangzhou (the capital of Zhejiang Province). All samples were collected within 30 km of QNPP during the period of 2012–2019 (Fig. 1), and the detailed sampling information is listed in Table 1.

**Table 1:** Samples collected from QNPP in this work during 2012–2019.

Sample category	Type or species	Location (coordinates)	Distance from QNPP (km)	Sampling frequency and time	Sample size	No. of subsamples
Fallout	Total atmospheric deposition	30° 31′ 4″ (N) 120° 55′ 40″ (E)	9.8	Quarterly End of every month	10 L	3
Water	Surface water	30° 32′ 56″ (N) 120° 49′ 48″ (E)	17.2	Twice per year May, October	50 L	3
	Tap water	30° 31′ 4″ (N) 120° 55′ 40″ (E)	9.8	Twice per year May, October	50 L	3
Food	Mullet	30° 33′ 57″ (N) 120° 1′ 9″ (E)	15.0	Annually May	10 kg	3
	Rice	30° 24′ 20″ (N) 120° 46′ 52″ (E)	18.8	Annually November	20 kg	3
	Salsola	30° 25′ 41″ (N) 120° 57′ 22″ (E)	1.8	Annually June	10 kg	3
	Crucian carp	30° 32′ 19″ (N) 120° 42′ 49(E)	26.9	Annually August	10 kg	3

Total (dry and wet) atmospheric deposition samples were monitored quarterly at a location 9.8 km away from QNPP. A total deposition collector (ZJC-VI deposition automatic collector, Zhejiang Hengda technology co.) with a surface area of 0.25 m^2^ was placed on the open space of a building roof at Center for Disease Control and Prevention of Haiyan for sample collection. The sample was collected at the end of each month and bulked quarterly for radioactivity measurement.

Surface waters were collected in Qianmudang reservoir, and tap waters were collected at the same place as for the total atmospheric deposition samples. The water samples were collected twice per year from each location in May (wet season) and October (dry season), respectively.

As the most popular food for Haiyan residents, mullet, salsola, rice and crucian carp were collected annually in this work for radioactivity monitoring. Mullet and salsola were locally produced in Haiyan, whereas rice and crucian carp were collected in the Haining City (the primary supplier of rice and crucian carp to local Haiyan population).

### Reagents and standards

Anhydrous alcohol, sulfuric acid, acetone, hydrochloric acid, hydrogen peroxide, nitric acid, oxalic acid, ammonia, and di (2-ethylhexyl) phosphoric acid used for this study were all analytically grade. A ^90^Sr-^90^Y standard solution (9.78 Bq/g of ^90^Sr in 0.1 mol/L nitric acid) and a ^137^Cs standard solution (1.47 Bq/g of ^137^Cs in 0.1 mol/L nitric acid) were purchased from National Institute of Metrology, China. Electroplating source of ^90^Sr-^90^Y with radioactivity intensity (Number of particles on the surface/2*π*·min) of 1.20 × 10^3^ was used for calibrating the detection efficiency of the instrument.

### Sample preparation and measurement

All dried food samples were placed in a 10 L quartz crucible and ashed with a microwave-ashing furnace (MKX-R4HB, Qingdao Maikewei Microwave Technology Co. Ltd) according to the rapid pretreatment method^11^ with gradual temperature increase from 100–150 °C to 500 °C (Table S1 in supporting information). All total atmospheric deposition samples were dried on a graphite heating plate (YKM-400C; Changsha Yonglekang Instrument Equipment Co., Ltd.) at 100℃ and then ashed in a muffle furnace (Thermo Fisher Scientific Co., Ltd.) at 450℃ for 8 h.

The radioachemical analysis of ^90^Sr in water and ash samples was according to the Chinese national standard procedures^12–15^. In general, Sr (100 mg) and Y (20 mg) carriers were added to water (adjusted to pH = 1.0 with concentrated HNO_3_) and ash samples. For water samples (50 L of each), SrCO_3_/CaCO_3_ precipitation was used to pre-concentrate Sr. The SrCO_3_/CaCO_3_ precipitate was dissolved with 6 mol/L HNO_3_ and the sample was adjusted to pH = 1 for further chromatographic purification.

For ash samples (5–30 g), after treated with 3–10 mL of concentrated HNO_3_ and 3 mL of H_2_O_2_, and evaporated to dryness, a second ashing was performed at 600 ℃ to ensure the complete decomposition of organic substances. Each ash sample was digested with 30–83 mL of 6 mol/L HCl for two times, the combined leachate was proceeded with CaC_2_O_4_/SrC_2_O_4_ precipitation for Sr pre-concentration. The CaC_2_O_4_/SrC_2_O_4_ precipitate was ashed in a muffle furnace (600℃, 1 h), and dissolved in 1.5 mol/L HNO_3_ for further chromatographic purification.

The processed sample solution for water or ash was loaded onto a chromatographic column (1 cm diameter × 15 cm length) containing di-(2-ethylhexyl) phosphoric acid (HDEHP), at a flow rate of 2 ml/min to separate ^90^Y. The column was washed with 40 mL of 1.5 mol/L HNO_3_ at 2 ml/min, and Y was eluted with 30 mL of 6 mol/L HNO_3_ at 1 ml/min. The separation time was recorded to calibrate the decay of ^90^Y. Yttrium was finally prepared as oxalate precipitation for gravimetric measurement of Y chemical yields prior to detection with a low background α/β counter (LB790; German Berthold Technology Company). Each sample was counted for 10 cycles, with each cycle for 100 min^12–15^.

The determination of ^137^Cs in water and ash samples was according to Chinese national standards^16–19^. Prior to ^137^Cs determination, the samples were kept for one month to allow for a complete decay of ^131^I. Each dried food sample was screened with gamma spectrometry to eliminate the interferences of other short-lived radiocesium (^134^Cs, ^136^Cs, and ^138^Cs) on ^137^Cs measurement by beta counting. Cesium carrier (20 mg) was added to the water (50 L, adjusted with nitric acid to pH = 2.0) and ash samples (5–20 g). Cesium contained in each ash sample was leached with 1.5 mol/L HNO_3_ for several times. Ammonium phosphomolybdate (AMP) was added to water or leachate sample (from the ash sample) to adsorb cesium. The precipitate was filtered and dissolved in NaOH solution. Cesium was finally separated as Cs_3_Bi_2_I_9_ precipitate in citric acid and acetic acid medium for gravimetric determination of chemical yield and measurement of ^137^Cs using the low background α/β counter. The measurement time was kept 100 min per cycle, in total of 10 cycles for each sample^16–19^. All the analytical results obtained in this work were summarized in Tables S2, S3, S4 in the supporting information.

## Results and discussion

### ^90^Sr and ^137^Cs activity concentrations in total atmospheric deposition

**Figure 2:**
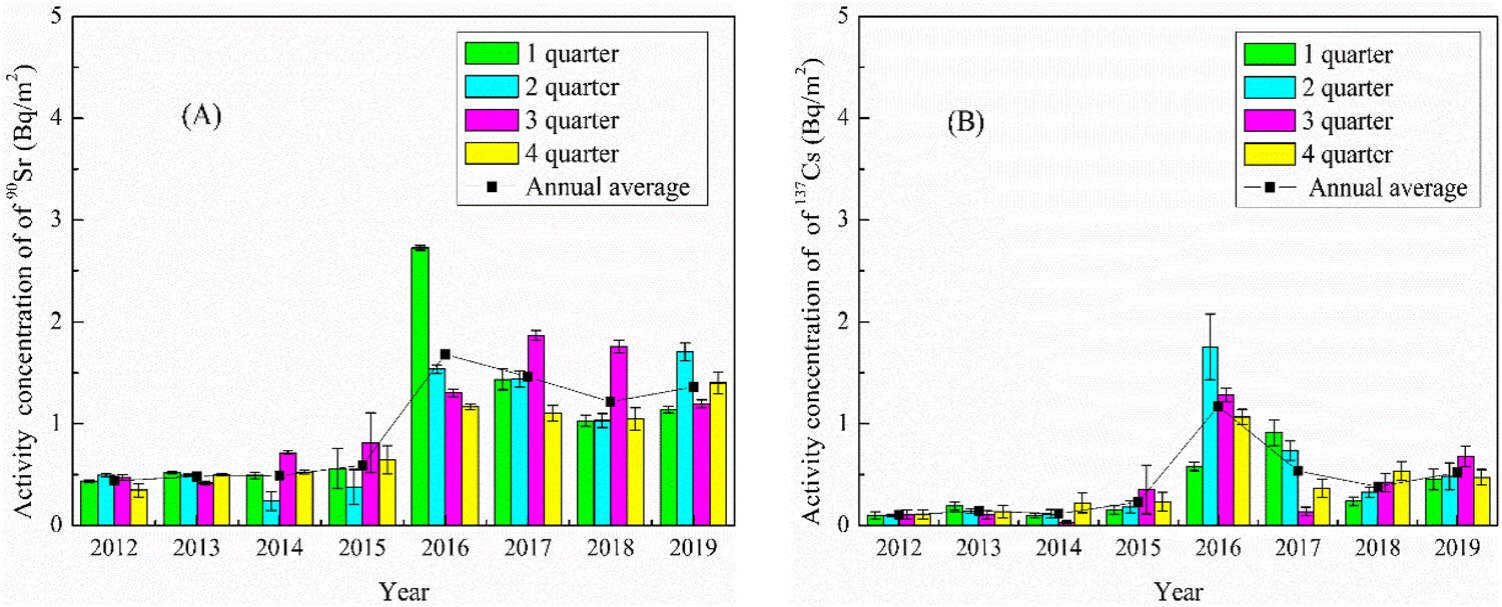
Variation of ^90^Sr (**A**) and ^137^Cs (**B**) activity concentration in total atmospheric deposition collected around of QNPP during 2012–2019.

The ^90^Sr and ^137^Cs activity concentrations in total atmospheric deposition are widely used indicators for revealing potential radioactive pollution in the atmosphere. The inter-annual and seasonal variations in activity concentrations of ^90^Sr and ^137^Cs in total atmospheric deposition collected around QNPP during the period of 2012–2019 are presented in Fig. 2. The observed annual average activity concentrations range within 0.44–1.68 Bq/m^2^ (mean ± sd = 0.97 ± 0.51 Bq/m^2^) for ^90^Sr and 0.10–1.17 Bq/m^2^ (mean ± sd = 0.40 ± 0.36 Bq/m^2^) for ^137^Cs, respectively. ^90^Sr and ^137^Cs activity concentrations demonstrate similar inter-annual variabilities across the entire study period, with the lowest annual average constantly obtained in 2012–2015 and the highest values in 2016 followed by a gradual decreasing trend until 2019. The appearance of peak ^90^Sr and ^137^Cs activity concentrations in 2016, which are up to ca. 4 and 11 times higher compared to the other years, respectively, potentially indicates additional radioactive plume in the study region in 2016 (see discussion later).

No clear seasonal variations of ^90^Sr and ^137^Cs in total atmospheric deposition were observed in this study. Though the highest activity concentrations of ^90^Sr and ^137^Cs both occurred in 2016, ^90^Sr peaked in the first quarter of 2016 whereas ^137^Cs peaked in the second quarter of 2016. This might be associated to the different source input functions and transport processes between ^137^Cs and ^90^Sr. ^137^Cs is particle reactive and readily attached to aerosols and dust, whereas ^90^Sr is more water soluble and easier to be dissolved in rainwater^20^. The late occurrence of ^137^Cs peak compared to ^90^Sr peak in 2016 could be due to the association of ^137^Cs to fine particles suspended in the atmosphere which prolonged the residence time of ^137^Cs in the atmosphere; and the plum rain in the second quarter (mostly in May) of 2016 flushed the fine particles and thereby prompted the deposition of ^137^Cs onto the ground. Nevertheless, both ^90^Sr and ^137^Cs activity concentrations in the atmosphere around QNPP were generally low in the past decade, and can be considered as baseline concentrations. This low background presents an opportunity to observe any future changes in the ^90^Sr and ^137^Cs concentrations and therefore can serve as baseline data for any future inputs from QNPP and other potential nuclear activities.

### ^90^Sr and ^137^Cs activity concentrations in water samples

**Figure 3:**
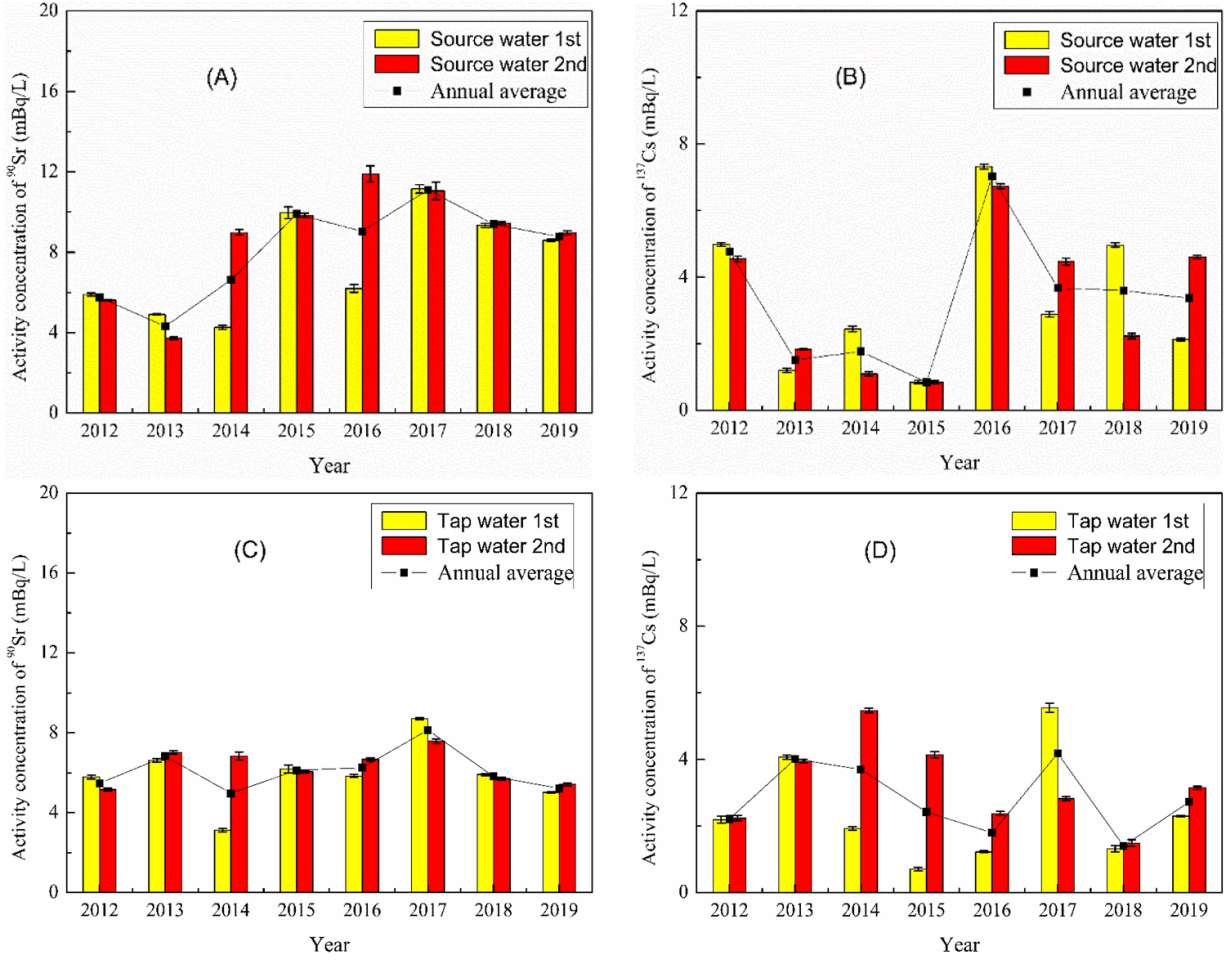
Variation of ^90^Sr and ^137^Cs activity concentration in source and tap water collected around QNPP during wet (1st) and dry (2nd) seasons in 2012–2019.

The time-series of activity concentrations of ^90^Sr and ^137^Cs in water samples collected around QNPP during the period of 2002–2019 are plotted in Fig. 3. Except for a few cases, no significant differences are observed for ^90^Sr and ^137^Cs activity concentrations in the two type waters between May and October in each year. ^90^Sr and ^137^Cs annual average activity concentrations in tap water (5.0–8.2 mBq/L for ^90^Sr and 1.4–4.2 mBq/L for ^137^Cs) are relatively stable compared to those in source water (4.3–11.1 mBq/L for ^90^Sr and 0.9–7.0 mBq/L for ^137^Cs) during 2012–2019. This should be a consequence of the additional water treatment process (e.g., filtration, disinfection) from raw water to tap water.

The highest ^90^Sr and ^137^Cs activity concentrations in source water are observed in 2016, with ^137^Cs in May and ^90^Sr in October, respectively. Whereas the peak values for both radionuclides in tap water, as observed in May 2017, seem to appear one year later compared to the source water. This again reflects the human intervention in the water treatment and delivery process. It is also noted that the annual average of ^137^Cs activity concentration in source water decreased rapidly after 2016 to reach similar level as in 2012, while ^90^Sr activity concentration was decreasing slowly and still nearly two times higher than the level in 2012. All the measured values for ^90^Sr and ^137^Cs activity concentrations in water samples around QNPP during 2012–2019 were below the concentration limits recommended by WHO and Chinese national standards^21,22^ and also comparable to the reported values for waters collected around other NPPs in China (Table 2).

**Table 2:** Activity concentrations of ^90^Sr and ^137^Cs in water and food samples collected around NPPs in various regions of China.

Samples		Tianwan NPP^23^ (sampling time: 2015–2016)	Haiyang NPP^24^ (sampling time: 2010–2011)	Sanmen NPP^25,26^ (sampling time: 2012–2019)	Qinshan NPP (this work)	Chinese regulatory limit^21,27^	WHO regulatory limit^22^
^90^Sr (mBq/L)	Source water	–	2.43–6.47	1.6–9.8	4.3–11.1	100	1000
	Tap water	–	1.21–9.00	1.2–8.3	5.0–8.2	100	1000
^137^Cs (mBq/L)	Source water	–	–	0.2–8.1	0.9–7.0	10	1000
	Tap water	–	–	0.7–5.1	1.4–4.2	10	1000
^90^Sr (Bq/kg)*	Rice	< 0.007	–	0.04–0.08	0.04–0.5	96	–
	Salsola	0.02–0.4	–	0.07–0.4	0.3–1.1	77	–
	Mullet	0.03–0.05	–	0.2–0.7	0.4–1.1	290	–
	Crucian carp	0.002–0.05	–	0.3–1.2	0.6–1.3	290	–
^137^Cs (Bq/kg)*	Rice	0.004–0.008	–	0.02–0.081	0.03–0.09	260	–
	Salsola	0.14–0.17	–	0.01–0.07	0.02–0.07	210	–
	Mullet	0.01–0.05	–	0.01–0.03	0.04–0.3	800	–
	Crucian carp	0.01–0.02	–	0.01–0.02	0.04–0.6	800	–

– means there was no relevant data.

*Results for food samples were given as Bq/kg fresh weight.

### ^90^Sr and ^137^Cs activity concentrations in food samples

**Figure 4:**
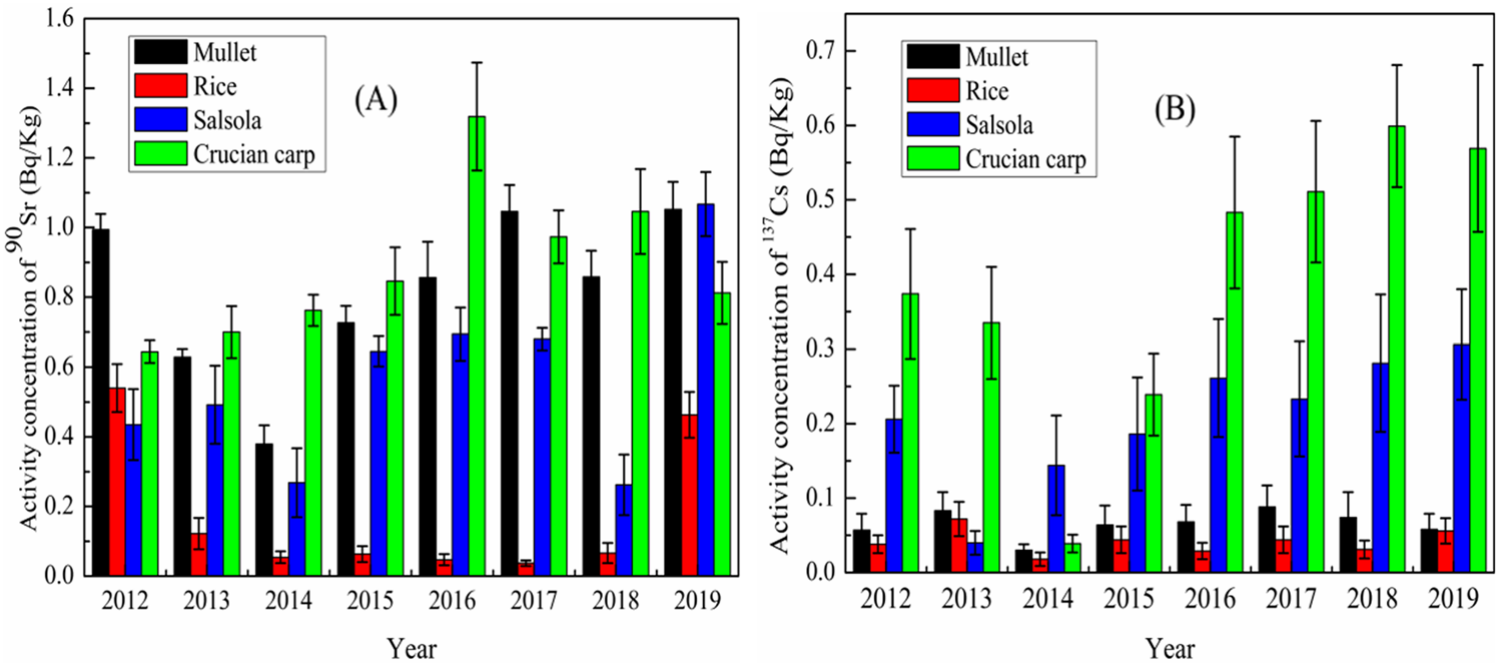
Activity concentrations of ^90^Sr (**A**) and ^137^Cs (**B**) in different food samples (mullet, rice, salsola, and crucian carp) collected around QNPP during 2012–2019.

The activity concentrations of ^90^Sr and ^137^Cs in different types of food samples collected around QNPP during 2012–2019 are presented in Fig. 4. Activity concentrations in rice, salsola, mullet and crucian carp were in the ranges of 0.04–0.5 Bq/kg fresh weight (f. w.), 0.3–1.1 Bq/kg f. w., 0.4–1.1 Bq/kg f. w. and 0.6–1.3 Bq/kg f. w. for ^90^Sr, and 0.03–0.09 Bq/kg f. w., 0.02–0.07 Bq/kg f. w., 0.04–0.3 Bq/kg f. w, and 0.04–0.6 Bq/kg f. w. for ^137^Cs, respectively. Our results are comparable to the activity concentrations of ^90^Sr and ^137^Cs in similar samples collected from other different NPPs in China (Table 2), and far below the Chinese regulatory limit for general foodstuffs.

Though no general trends in the temporal variations of ^90^Sr and ^137^Cs activity concentrations in the four food species can be seen during 2012–2019, the lowest activity concentrations for both radionuclides are mostly observed in rice and the highest values in crucian carp. Interestingly, ^90^Sr activity concentration is often higher in mullet compared to salsola, whereas the opposite is obtained for ^137^Cs. Even between the two fish species, the difference in ^137^Cs activity concentration (up to 10 times) is much higher than that in ^90^Sr activity concentration (max. 1.5 times). These features are linked to ^90^Sr and ^137^Cs levels in the environment where different biota are growing and their different physiological mechanisms for the incorporation and excretion processes of Sr and Cs^28,29^. For example, mullet inhabits salt water and brackish waters whereas crucian carp is a freshwater fish that inhabits lakes, ponds, and slow-moving rivers.

In the freshwater systems, the high particle/colloid loads may effectively scavenge ^137^Cs, whereas ^90^Sr is kept stable in the water due its highly conservative behavior. Freshwater in some rivers has been found to include high concentrations of ^90^Sr^30,31^. Besides, higher salinity in brackish water may facilitate the uptake of ^137^Cs (similar to potassium) into the fish. The values of concentration factors for Cs and Sr in marine fishes are 100 and 3, respectively^32^. Therefore, it is reasonable to expect a larger difference in ^137^Cs activity concentration between mullet and crucian carp compared to that in ^90^Sr activity concentration.

The annual effective dose (*AED*) due to the ingestion of ^137^Cs and ^90^Sr in foods (i.e., mullet, salsola, rice and crucian carp) were estimated based on the Chinese national standard^33^ using the following equation:

1 $$AED = \, (I_{Cs} + I_{Sr} ) \cdot e(g)$$

where *I*_*Cs*_ and *I*_*Sr*_ is the annual intake of ^137^Cs and ^90^Sr in foods (Bq/y), respectively. *e*(*g*) was the dose conversion coefficient of ingested radionuclide ^137^Cs and ^90^Sr in foods. Dose conversion coefficients of 1.3 × 10^–8^ Sv/Bq for ^137^Cs and 2.8 × 10^–8^ Sv/Bq for ^90^Sr were used in this study according to the Chinese national standards .

The obtained *AED* through ingestion of ^137^Cs and ^90^Sr in foods for the local population around QNPP was calculated to be in the range of 1.3 × 10^–4^–1.1 × 10^–3^ Sv/y during 2012–2019, with a mean value of (3.9 ± 4.0) × 10^–4^ Sv/y. In this calculation, the annual per capita human food consumption (0.19, 0.085, 67.84 and 0.40 kg/year/person for mullet, salsola, rice and crucian carp, respectively) by the local public were according to the survey in 2019 and assumed to be constant during 2012–2019. The estimated mean *AED* for these eight years is considered negligible in respect to the recommended limit (1 mSv/y) established by International Commission on Radiological Protection (ICRP). This reveals that the foods around QNPP were at a safe level during the period 2012–2019.

### Source term identification via ^137^Cs/^90^Sr activity ratio

**Figure 5:**
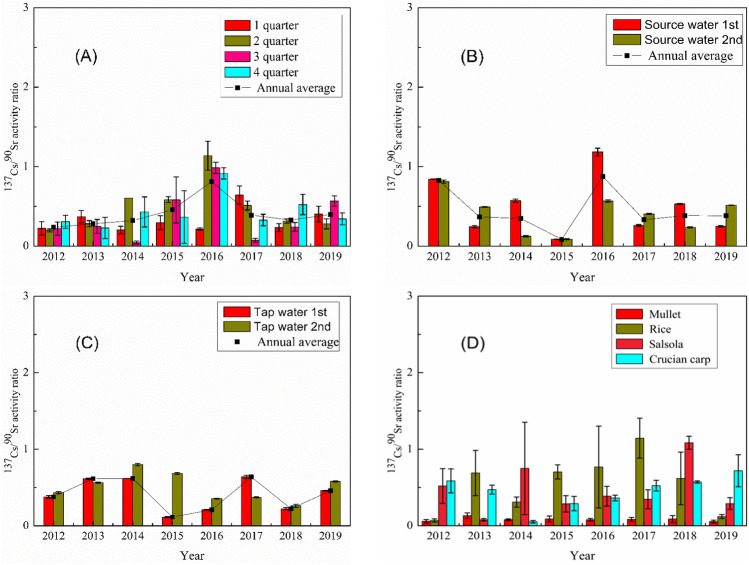
^137^Cs/^90^Sr activity ratio in total atmospheric deposition (**A**), source water (**B**), tap water (**C**) and food samples (**D**) during 2012–2019.

The temporal variations of ^137^Cs/^90^Sr activity ratios in the investigated samples during 2012–2019 are presented in Fig. 5. For total atmospheric deposition, the annual average ^137^Cs/^90^Sr activity ratios ranged from 0.24 to 0.81, with notable peaks observed in 2016. Narrower distribution ranges of ^137^Cs/^90^Sr activity ratios were observed in the water and food samples compared to the total atmospheric deposition, varying within 0.085–1.18 for source water, 0.11–0.80 for tape water, 0.055–0.13 for mullet, 0.069–1.14 for rice, 0.078–1.08 for salsola, 0.052–0.72 for crucian carp, respectively.

The background ^137^Cs and ^90^Sr levels on the world surface are mostly due to global fallout of atmospheric nuclear weapons testing during 1945–1980. The activity ratio of ^137^Cs/^90^Sr from the global fallout deposition was estimated to be about 1.6^34,35^. Our results indicate that all the ^137^Cs/^90^Sr activity ratios are lower than the global fallout signal. This might be associated to faster removal of ^137^Cs compared to ^90^Sr originated from global fallout and/or additional input of ^90^Sr in the surrounding environment of the study region. The latter is very unlikely, because ^90^Sr concentrations measured in this work are in the background level and there is no record of significant radioactive releases in the study region. Besides, as any potential release of ^90^Sr (such as civil nuclear industry) would normally be accompanied by ^137^Cs release and often with much higher ^137^Cs/^90^Sr activity ratios compared to global fallout, owing to the lower volatility of Sr than Cs^36^. For example, very high ^137^Cs/^90^Sr ratios have been reported in the atmospheric fallout from Fukushima accident (∼1000)^37^ and Chernobyl accident (∼250)^38^. In seawater, ^137^Cs/^90^Sr ratios reached 39 ± 1 beyond the coast of Japan due to massive liquid releases of cooling water in spring 2011^37^. The ^137^Cs/^90^Sr activity ratios before the Fukushima accident were reported in the range of 2.6–18 in Japanese fish whereas the ^137^Cs/^90^Sr activity ratios ranged from 98 to 480 after the accident^39^.

As ^137^Cs is more readily adsorbed and immobilized on clay minerals while ^90^Sr exhibits a higher mobility, it is reasonable to foresee a decrease in global fallout derived ^137^Cs/^90^Sr activity ratios in some freshwater and food samples. This has been approved in earlier studies where activity ratios of ^137^Cs/^90^Sr in wheat and polished rice from Japan increased by nearly 20 and 3 times, respectively, from 1959 to 1995^40^.

^137^Cs and ^90^Sr in the dry atmospheric deposition was reported to reflect their signature in the ground-level air, which is mostly from resuspension of the deposited ^137^Cs and ^90^Sr in soil^41^. Sr is chemically easier to elute than Cs in the soil column by rainwater^20^, therefore ^137^Cs/^90^Sr activity ratio in the typical surface soil usually becomes higher than the typical global fallout value^30,31^. However, lower ^137^Cs/^90^Sr activity ratios compared to the global fallout level were observed in the total atmospheric deposition in this work. This might be related to the relative high annual precipitation rate in the study region, therefore the total atmospheric deposition signal are predominated by wet precipitation associated to lower ^137^Cs/^90^Sr activity ratios than global fallout, but are comparable to those measured in fresh waters in this work.

The peak ^137^Cs/^90^Sr activity ratio (1.14 ± 0.18) in the total atmospheric deposition in the second quarter of 2016 was ca. 2.5 times higher than the average values (0.46 ± 0.15) in 2015. This might suggest an additional radioactive input with higher ^137^Cs/^90^Sr activity ratio in the study region in 2016. As a potential consequence of this additional radioactive source, maximum ^137^Cs/^90^Sr activity ratios were observed in the source water (1.18 ± 0.05) in 2016, rice (1.14 ± 0.26) in 2017 and salsola (1.08 ± 0.09) in 2018, respectively. The occurrence of peak ^137^Cs/^90^Sr activity ratio following the sequence of atmosphere-water-biota pinpoints the additional radioactive source might be a direct atmospheric fallout either from local nuclear facilities (e.g., QNPP and other local NPPs) or global sources (e.g., Fukushima accident and others).

It is virtually impossible that the Fukushima fallout arrived to the study region in 2016, five years after the accident. At the first several days of the accidents, air transport in the mid-latitudes was dominated by prevailing westerly winds, which could circle around the globe in 2 weeks^42^. For example, several pulses of radioactive emission from Fukushima were observed in Northern Taiwan 14 days after the accident^43^. We suspect the potential additional radioactive input in 2016 is from a local source. It was reported that the two units in Fangjiashan NPP (FJSNPP) as an expansion of Phase I in QNPP, were put into operation in December 2014 and February 2015, respectively^10^. The commencement of the two units could potentially introduce increased release of ^137^Cs and ^90^Sr to some extent. However, this does not support the decrease of ^137^Cs and ^90^Sr concentrations and ^137^Cs/^90^Sr activity ratios in the total deposition for the following years. Therefore, further confirmation is needed due lack of operational and discharges data from QNPP in this work.

## Conclusions

This study presents the first long-term systematic study of levels, variations and sources of ^90^Sr and ^137^Cs in environmental and food samples around QNPP in 2012–2019. The concentrations of ^90^Sr and ^137^Cs obtained in this work represent the background level, with all the values below the recommendations by WHO and Chinese national standard. Moreover, the peak concentrations of ^90^Sr and ^137^Cs appeared in 2016 were suspected to be related with an additional input from the local facility, but it requires further confirmation. This study indicate the high sensitivity of ^90^Sr and ^137^Cs, especially the ^137^Cs/^90^Sr activity ratio for detecting any radioactive release in the region. In the future, ^90^Sr and ^137^Cs monitoring is recommended as regional safeguard measure against accidental release from the local nuclear power plant.

## Supplementary Information

Supplementary Information.
