# Supplementary material for: Long-term variation of 90Sr and 137Cs in environmental and food samples around Qinshan nuclear power plant, China
Source: Sci Rep. 2021 Oct 22;11:20903. doi: 10.1038/s41598-021-00114-y (PMC8536726; doi:10.1038/s41598-021-00114-y)
Supplement: Supplementary file 1 — Supplementary Information. [file 41598_2021_114_MOESM1_ESM.docx]

**Supporting information**

**Long-term variation of ^90^Sr and ^137^Cs in environmental and food samples around Qinshan Nuclear Power Plant, China**

Yiyao Cao^1^, Zhixin Zhao^2^, Peng Wang^1^, Shunfei Yu^1^, Zhongjun Lai^1^, Meibian Zhang^3^, Xiangjing Gao^1^, Yaoxian Zhao^1^, Zhiqiang Xuan^1^, Hong Ren^1^, Dongxia Zhang^1^, Xiaoming Lou^1,*^

^1^ Department of Occupational Health and Radiation Protection, Zhejiang Provincial Center for Disease Control and Prevention, Hangzhou 310051, Zhejiang, China

^2^ Hangzhou Hospital for the Prevention and Treatment of Occupational Disease, Hangzhou 310051, Zhejiang, China

^3^ National Institute of Occupational Health and Poison Control, Chinese Center for Disease Control and Prevention, Beijing 100050, China.

*Correspondence: Xiaoming Lou

Zhejiang Provincial Center for Disease Control and Prevention, Zhejiang, China.

Tel./Fax: +86-0571-87115089

E-mail: 540040349@qq.com

**Summary**

The supporting information includes four tables summarizing the programmed heating conditions for microwave-assisted ashing of food samples in this work (Table S1), ^137^Cs and ^90^Sr activity concentrations and ^137^Cs/^90^Sr activity ratios in total atmospheric deposition (Table S2), source and tap waters (Table S3) and food samples (Table S4) around Qinshan Nuclear power plant (QNPP) during 2012–2019.

**Table S1** Programmed heating conditions for microwave-assisted ashing of food samples in this work

| Food | Temperature（℃） | Duration（h） |
| --- | --- | --- |
| Salsola | 98 | 0.5 |
|  | 240 | 3 |
|  | 320 | 1 |
|  | 500 | 4 |
| Crucian carp  and  Mullet | 127 | 2 |
|  | 230 | 10 |
|  | 350 | 2 |
|  | 500 | 12 |
| Rice | 150 | 12 |
|  | 270 | 12 |
|  | 400 | 5 |
|  | 500 | 5 |

**Table S2** ^137^Cs and ^90^Sr activity concentrations and ^137^Cs/^90^Sr activity ratios in total atmospheric deposition during 2012–2019

| **Year** |  | **1^st^ Quater** | | | **2^nd^ Quater** | | | **3^rd^ Quater** | | | **4^th^ Quater** | | | **Annual average** | | |
| --- | --- | --- | --- | --- | --- | --- | --- | --- | --- | --- | --- | --- | --- | --- | --- | --- |
|  |  | **^90^Sr** | **^137^Cs** | **^137^Cs/^90^Sr** | **^90^Sr** | **^137^Cs** | **^137^Cs/^90^Sr** | **^90^Sr** | **^137^Cs** | **^137^Cs/^90^Sr** | **^90^Sr** | **^137^Cs** | **^137^Cs/^90^Sr** | **^90^Sr** | **^137^Cs** | **^137^Cs/^90^Sr** |
|  |  | **Bq/m^2^** | **Bq/m^2^** |  | **Bq/m^2^** | **Bq/m^2^** |  | **Bq/m^2^** | **Bq/m^2^** |  | **Bq/m^2^** | **Bq/m^2^** |  | **Bq/m^2^** | **Bq/m^2^** |  |
| **2012** | **value** | 0.435 | 0.098 | 0.222 | 0.496 | 0.098 | 0.197 | 0.471 | 0.105 | 0.219 | 0.348 | 0.109 | 0.306 | 0.438 | 0.102 | 0.236 |
|  | **sd** | 0.012 | 0.039 | 0.085 | 0.017 | 0.012 | 0.019 | 0.029 | 0.047 | 0.085 | 0.067 | 0.043 | 0.083 | 0.064 | 0.006 | 0.048 |
| **2013** | **value** | 0.518 | 0.192 | 0.370 | 0.495 | 0.140 | 0.282 | 0.415 | 0.104 | 0.246 | 0.502 | 0.137 | 0.277 | 0.483 | 0.143 | 0.294 |
|  | **sd** | 0.009 | 0.043 | 0.078 | 0.013 | 0.022 | 0.038 | 0.018 | 0.042 | 0.089 | 0.012 | 0.063 | 0.135 | 0.046 | 0.036 | 0.053 |
| **2014** | **value** | 0.491 | 0.099 | 0.202 | 0.239 | 0.112 | 0.603 | 0.717 | 0.032 | 0.045 | 0.522 | 0.223 | 0.430 | 0.492 | 0.116 | 0.320 |
|  | **sd** | 0.022 | 0.022 | 0.048 | 0.091 | 0.043 | 0.359 | 0.014 | 0.013 | 0.019 | 0.017 | 0.097 | 0.189 | 0.196 | 0.079 | 0.246 |
| **2015** | **value** | 0.558 | 0.154 | 0.294 | 0.378 | 0.181 | 0.585 | 0.812 | 0.354 | 0.580 | 0.645 | 0.233 | 0.365 | 0.598 | 0.231 | 0.456 |
|  | **sd** | 0.196 | 0.042 | 0.084 | 0.172 | 0.048 | 0.240 | 0.241 | 0.239 | 0.518 | 0.139 | 0.092 | 0.139 | 0.181 | 0.088 | 0.149 |
| **2016** | **value** | 2.726 | 0.581 | 0.213 | 1.537 | 1.754 | 1.137 | 1.305 | 1.282 | 0.984 | 1.169 | 1.067 | 0.914 | 1.684 | 1.171 | 0.812 |
|  | **sd** | 0.023 | 0.043 | 0.016 | 0.042 | 0.322 | 0.181 | 0.037 | 0.066 | 0.070 | 0.029 | 0.074 | 0.071 | 0.711 | 0.487 | 0.410 |
| **2017** | **value** | 1.435 | 0.912 | 0.642 | 1.439 | 0.735 | 0.511 | 1.870 | 0.137 | 0.074 | 1.103 | 0.365 | 0.328 | 1.462 | 0.537 | 0.389 |
|  | **sd** | 0.102 | 0.129 | 0.116 | 0.082 | 0.095 | 0.056 | 0.048 | 0.041 | 0.024 | 0.075 | 0.091 | 0.072 | 0.314 | 0.351 | 0.246 |
| **2018** | **value** | 1.030 | 0.239 | 0.234 | 1.031 | 0.327 | 0.315 | 1.759 | 0.422 | 0.241 | 1.047 | 0.535 | 0.523 | 1.217 | 0.381 | 0.328 |
|  | **sd** | 0.056 | 0.043 | 0.050 | 0.069 | 0.052 | 0.029 | 0.063 | 0.088 | 0.055 | 0.112 | 0.092 | 0.128 | 0.362 | 0.127 | 0.135 |
| **2019** | **value** | 1.140 | 0.456 | 0.402 | 1.706 | 0.481 | 0.279 | 1.196 | 0.679 | 0.566 | 1.402 | 0.473 | 0.342 | 1.361 | 0.522 | 0.397 |
|  | **sd** | 0.034 | 0.104 | 0.100 | 0.088 | 0.132 | 0.064 | 0.039 | 0.099 | 0.065 | 0.108 | 0.076 | 0.076 | 0.256 | 0.105 | 0.123 |

**Table S3** ^137^Cs and ^90^Sr activity concentrations and ^137^Cs/^90^Sr activity ratios in source and tap water during 2012–2019

| **Year** |  | **1^st^ source water** | | | **2^nd^ source water** | | | **Source water annual average** | | | **1^st^ tap water** | | | **2^nd^ tap water** | | | **Tap water annual average** | | |
| --- | --- | --- | --- | --- | --- | --- | --- | --- | --- | --- | --- | --- | --- | --- | --- | --- | --- | --- | --- |
|  |  | **^90^Sr** | **^137^Cs** | **^137^Cs/^90^Sr** | **^90^Sr** | **^137^Cs** | **^137^Cs/^90^Sr** | **^90^Sr** | **^137^Cs** | **^137^Cs/^90^Sr** | **^90^Sr** | **^137^Cs** | **^137^Cs/^90^Sr** | **^90^Sr** | **^137^Cs** | **^137^Cs/^90^Sr** | **^90^Sr** | **^137^Cs** | **^137^Cs/^90^Sr** |
|  |  | **Bq/L** | **Bq/L** |  | **Bq/L** | **Bq/L** |  | **Bq/L** | **Bq/L** |  |  |  |  |  |  |  | **Bq/L** | **Bq/L** |  |
| **2012** | **value** | 5.902 | 4.984 | 0.844 | 5.617 | 4.554 | 0.811 | 5.760 | 4.769 | 0.828 | 5.788 | 2.186 | 0.378 | 5.160 | 2.238 | 0.434 | 5.474 | 2.212 | 0.406 |
|  | **sd** | 0.071 | 0.045 | 0.003 | 0.045 | 0.089 | 0.020 | 0.202 | 0.304 | 0.024 | 0.101 | 0.105 | 0.016 | 0.065 | 0.076 | 0.013 | 0.444 | 0.036 | 0.040 |
| **2013** | **value** | 4.904 | 1.198 | 0.244 | 3.726 | 1.829 | 0.491 | 4.315 | 1.514 | 0.368 | 6.617 | 4.071 | 0.615 | 7.021 | 3.953 | 0.563 | 6.819 | 4.012 | 0.589 |
|  | **sd** | 0.033 | 0.066 | 0.014 | 0.048 | 0.024 | 0.005 | 0.833 | 0.446 | 0.174 | 0.092 | 0.065 | 0.009 | 0.075 | 0.052 | 0.007 | 0.286 | 0.084 | 0.037 |
| **2014** | **value** | 4.263 | 2.439 | 0.572 | 8.987 | 1.098 | 0.122 | 6.625 | 1.769 | 0.347 | 3.120 | 1.928 | 0.618 | 6.839 | 5.472 | 0.801 | 4.979 | 3.700 | 0.709 |
|  | **sd** | 0.099 | 0.087 | 0.019 | 0.135 | 0.065 | 0.009 | 3.340 | 0.948 | 0.318 | 0.079 | 0.053 | 0.001 | 0.211 | 0.067 | 0.015 | 2.630 | 2.506 | 0.129 |
| **2015** | **value** | 9.966 | 0.847 | 0.085 | 9.833 | 0.845 | 0.086 | 9.899 | 0.846 | 0.085 | 6.193 | 0.704 | 0.114 | 6.053 | 4.142 | 0.684 | 6.123 | 2.423 | 0.399 |
|  | **sd** | 0.296 | 0.054 | 0.003 | 0.102 | 0.049 | 0.005 | 0.094 | 0.002 | 0.001 | 0.207 | 0.055 | 0.005 | 0.063 | 0.092 | 0.012 | 0.099 | 2.431 | 0.403 |
| **2016** | **value** | 6.198 | 7.327 | 1.184 | 11.892 | 6.733 | 0.567 | 9.045 | 7.030 | 0.875 | 5.837 | 1.225 | 0.210 | 6.668 | 2.373 | 0.356 | 6.252 | 1.799 | 0.283 |
|  | **sd** | 0.191 | 0.073 | 0.048 | 0.399 | 0.072 | 0.014 | 4.026 | 0.420 | 0.436 | 0.078 | 0.028 | 0.003 | 0.082 | 0.063 | 0.005 | 0.587 | 0.812 | 0.103 |
| **2017** | **value** | 11.157 | 2.884 | 0.259 | 11.052 | 4.467 | 0.404 | 11.105 | 3.676 | 0.332 | 8.704 | 5.550 | 0.638 | 7.587 | 2.819 | 0.372 | 8.145 | 4.185 | 0.505 |
|  | **sd** | 0.217 | 0.082 | 0.011 | 0.433 | 0.098 | 0.007 | 0.074 | 1.119 | 0.103 | 0.059 | 0.133 | 0.020 | 0.113 | 0.066 | 0.007 | 0.790 | 1.932 | 0.188 |
| **2018** | **value** | 9.346 | 4.965 | 0.531 | 9.447 | 2.233 | 0.236 | 9.396 | 3.599 | 0.384 | 5.915 | 1.318 | 0.223 | 5.707 | 1.489 | 0.261 | 5.811 | 1.403 | 0.242 |
|  | **sd** | 0.098 | 0.066 | 0.006 | 0.075 | 0.073 | 0.006 | 0.071 | 1.932 | 0.209 | 0.056 | 0.092 | 0.015 | 0.068 | 0.101 | 0.021 | 0.147 | 0.121 | 0.027 |
| **2019** | **value** | 8.586 | 2.131 | 0.248 | 8.972 | 4.607 | 0.513 | 8.779 | 3.369 | 0.381 | 5.022 | 2.298 | 0.458 | 5.423 | 3.145 | 0.580 | 5.223 | 2.722 | 0.519 |
|  | **sd** | 0.067 | 0.043 | 0.007 | 0.102 | 0.054 | 0.001 | 0.273 | 1.751 | 0.188 | 0.034 | 0.021 | 0.005 | 0.078 | 0.047 | 0.006 | 0.284 | 0.598 | 0.086 |

**Table S4** ^137^Cs and ^90^Sr activity concentrations and ^137^Cs/^90^Sr activity ratios in different food samples collected around QNPP during 2012–2019

| **Year** |  | **Mullet** | | | **Rice** | | | **Salsola** | | | **Crucian carp** | | |
| --- | --- | --- | --- | --- | --- | --- | --- | --- | --- | --- | --- | --- | --- |
|  |  | **^90^Sr** | **^137^Cs** | **^137^Cs/^90^Sr** | **^90^Sr** | **^137^Cs** | **^137^Cs/^90^Sr** | **^90^Sr** | **^137^Cs** | **^137^Cs/^90^Sr** | **^90^Sr** | **^137^Cs** | **^137^Cs/^90^Sr** |
|  |  | **Bq/kg f.w.** | **Bq/kg f.w.** |  | **Bq/kg f.w.** | **Bq/kg f.w.** |  | **Bq/kg f.w.** | **Bq/kg f.w.** |  | **Bq/kg f.w.** | **Bq/kg f.w.** |  |
| **2012** | **value** | 0.994 | 0.057 | 0.058 | 0.540 | 0.038 | 0.069 | 0.435 | 0.206 | 0.519 | 0.644 | 0.374 | 0.586 |
|  | **sd** | 0.045 | 0.022 | 0.022 | 0.069 | 0.012 | 0.021 | 0.102 | 0.045 | 0.227 | 0.032 | 0.087 | 0.157 |
| **2013** | **value** | 0.629 | 0.083 | 0.131 | 0.122 | 0.072 | 0.689 | 0.492 | 0.040 | 0.078 | 0.700 | 0.335 | 0.472 |
|  | **sd** | 0.023 | 0.025 | 0.037 | 0.045 | 0.023 | 0.295 | 0.112 | 0.016 | 0.016 | 0.074 | 0.075 | 0.059 |
| **2014** | **value** | 0.379 | 0.030 | 0.079 | 0.054 | 0.018 | 0.309 | 0.268 | 0.144 | 0.749 | 0.762 | 0.039 | 0.052 |
|  | **sd** | 0.054 | 0.008 | 0.011 | 0.017 | 0.009 | 0.066 | 0.099 | 0.067 | 0.603 | 0.045 | 0.012 | 0.017 |
| **2015** | **value** | 0.726 | 0.064 | 0.089 | 0.063 | 0.044 | 0.703 | 0.645 | 0.186 | 0.286 | 0.846 | 0.239 | 0.290 |
|  | **sd** | 0.049 | 0.026 | 0.038 | 0.023 | 0.018 | 0.092 | 0.043 | 0.076 | 0.107 | 0.097 | 0.055 | 0.094 |
| **2016** | **value** | 0.856 | 0.068 | 0.078 | 0.047 | 0.029 | 0.767 | 0.694 | 0.261 | 0.386 | 1.319 | 0.483 | 0.362 |
|  | **sd** | 0.103 | 0.023 | 0.017 | 0.016 | 0.011 | 0.535 | 0.076 | 0.079 | 0.129 | 0.155 | 0.102 | 0.037 |
| **2017** | **value** | 1.046 | 0.088 | 0.083 | 0.037 | 0.044 | 1.144 | 0.680 | 0.233 | 0.346 | 0.973 | 0.511 | 0.523 |
|  | **sd** | 0.076 | 0.029 | 0.025 | 0.008 | 0.018 | 0.261 | 0.032 | 0.077 | 0.124 | 0.076 | 0.095 | 0.070 |
| **2018** | **value** | 0.858 | 0.074 | 0.088 | 0.066 | 0.031 | 0.618 | 0.262 | 0.281 | 1.084 | 1.046 | 0.599 | 0.571 |
|  | **sd** | 0.075 | 0.034 | 0.045 | 0.029 | 0.012 | 0.343 | 0.087 | 0.092 | 0.085 | 0.122 | 0.082 | 0.015 |
| **2019** | **value** | 1.052 | 0.058 | 0.055 | 0.463 | 0.056 | 0.120 | 1.067 | 0.306 | 0.289 | 0.812 | 0.569 | 0.719 |
|  | **sd** | 0.079 | 0.021 | 0.018 | 0.066 | 0.017 | 0.026 | 0.092 | 0.074 | 0.078 | 0.089 | 0.112 | 0.209 |
